# Phase I results on the efficacy, safety and pharmacokinetics of lurbinectedin and irinotecan in advanced solid tumors

**DOI:** 10.1007/s10637-025-01583-y

**Published:** 2025-09-18

**Authors:** Alejandro Falcón, Santiago Ponce, Gregory M. Cote, Ana Gil, Jessica J. Lin, Bruno Bockorny, Julia Martínez, Carmen Kahatt, Sara Martinez, Pablo Zubiaur, Mariano Siguero, Martin Cullell-Young, Javier Jiménez, Jon Zugazagoitia, Luis Paz-Ares

**Affiliations:** 1 Servicio de Oncología Médica, Hospital Universitario Virgen del Rocio, Av. Manuel Siurot, S/N, 41013 Seville, Spain; 2 Hospital Universitario, 12 de Octubre, Madrid, Spain; 3 CNIO-H12O Lung Cancer Clinical Cancer Research Unit, Instituto de Investigación Biomédica I+12 & Centro Nacional de Investigaciones Oncológicas (CNIO), Madrid, Spain; 4 CIBERONC, Madrid, Spain; 5 Mass General Cancer Center, Boston, MA USA; 6 Beth Israel Deaconess Medical Center, Boston, MA USA; 7 Pharma Mar, Colmenar Viejo, Madrid, Spain; 8 Universidad Complutense, Madrid, Spain

**Keywords:** Lurbinectedin, Irinotecan, Phase I study

## Abstract

Lurbinectedin and irinotecan showed synergistic antitumor activity when combined in preclinical studies, and have non-completely overlapping toxicity profiles. A two-stage phase I/II trial was designed to evaluate the combination. The first (dose escalation) stage of the trial assessed two schedules, lurbinectedin on Day (D)1 plus irinotecan on D1,D8 or D1 every three weeks in 83 patients with relapsed advanced solid tumors. Two recommended doses (RDs) were defined for lurbinectedin on D1 plus irinotecan on D1,D8: lurbinectedin 2.0 mg/m^2^ plus irinotecan 75 mg/m^2^, and lurbinectedin 3.0 mg/m^2^ plus irinotecan 40 mg/m^2^, both with primary growth factor prophylaxis. No RD was defined for lurbinectedin on D1 plus irinotecan on D1. Lurbinectedin on D1 plus irinotecan on D1,D8 q3wk showed a manageable safety profile at the RDs, with most common toxicities being myelosuppression, fatigue and gastrointestinal disorders. No toxic deaths occurred. Thirteen confirmed partial responses and 24 disease stabilizations ≥ 4 months were found at all dose levels, including the RDs. Compared to other tumor types, antitumor activity was higher in small cell lung cancer (SCLC), soft tissue sarcoma (synovial), endometrial carcinoma, glioblastoma and pancreatic adenocarcinoma. No major pharmacokinetic interaction was found between lurbinectedin and irinotecan. The second (expansion) stage of the trial is evaluating the RD of lurbinectedin 2.0 mg/m^2^ plus irinotecan 75 mg/m^2^ with primary growth factor prophylaxis in selected advanced solid tumors. An ongoing phase III trial is also evaluating the combination in second-line SCLC after prior platinum-containing chemotherapy. Trial registration number: NCT02611024 (Nov 20, 2015).

**Supplementary Information:**

The online version contains supplementary material available at 10.1007/s10637-025-01583-y.

## Introduction

Lurbinectedin (Zepzelca®) inhibits oncogenic transcription primarily through binding to the exocyclic amino group of guanine-rich DNA sequences around promoters of protein-coding genes and evicting oncogenic transcription factors from their binding sites [1–3]. Lurbinectedin adducts induce the stalling of transcribing RNA polymerase II on the DNA and its specific ubiquitination and degradation, trigger the formation of DNA double-strand breaks, and induce apoptotic cell death [4, 5]. Lurbinectedin monotherapy has been approved in the U.S. and other countries for the treatment of adult patients with metastatic small cell lung cancer (SCLC) and disease progression on or after platinum-based chemotherapy [6].

Preclinical studies showed that lurbinectedin synergizes the antitumor effect of topoisomerase inhibitors. In vivo, the combination of lurbinectedin and irinotecan showed a synergistic antitumor effect in athymic mice bearing colon, non-small cell lung cancer (NSCLC), or pancreas xenografted tumors [7]. The toxicity profiles of lurbinectedin and irinotecan were not completely overlapping.

A phase I/II trial was designed to evaluate the lurbinectedin/irinotecan combination in patients with advanced solid tumors. The first part of the trial was a dose escalation stage to determine the recommended dose (RD) of the combination in patients with relapsed advanced solid tumors. This was followed by an expansion stage to evaluate the antitumor activity of the combination at the RD in tumor-specific cohorts selected based on the efficacy results of the first part. The safety profile and pharmacokinetics of the combination were evaluated throughout the trial.

The results of the dose escalation stage of the trial are described herein.

## Patients and methods

This open-label, non-randomized, uncontrolled study was divided into two parts. The first one was a phase I escalation stage with the primary objective of determining the maximum tolerated dose (MTD) and the RD of lurbinectedin combined with irinotecan in patients with selected advanced solid tumors using three different dose escalation schemes. Once the RD had been defined, the study would enter a phase II expansion stage with the primary objective of obtaining information on the clinical antitumor activity of the combination at the RD in specific tumor types, chosen based on the activity observed during escalation. Secondary objectives of both stages were to evaluate the safety profile and pharmacokinetics of the combination, and to detect potential major drug-drug interactions between lurbinectedin and irinotecan. No blinding or randomization were implemented.

### Eligibility criteria

Eligible patients in the escalation stage were aged ≥ 18 years; with Eastern Cooperative Oncology Group performance status (ECOG PS) score ≤ 1; life expectancy ≥ 3 months; advanced solid tumors; treated with no more than two prior lines of cytotoxic-containing chemotherapy for advanced disease; who had recovered from previous toxicities; and with adequate bone marrow, hepatic, renal and metabolic function.

Patients were excluded if they had been pretreated with lurbinectedin, trabectedin or topoisomerase I inhibitors; had received bone marrow or stem cell transplantation, or radiotherapy in > 35% of bone marrow; were lactating women or were not using effective contraceptives; or had symptomatic brain metastases or leptomeningeal disease, relevant cardiac disease, active infection, any disease interfering with study outcome, or hypersensitivity to lurbinectedin, irinotecan or any formulation component.

### Study treatment

Three escalating groups were evaluated in the escalation stage. Two groups evaluated a schedule of lurbinectedin on Day (D)1 every three weeks (q3wk) combined with irinotecan on D1,D8 q3wk: the lurbinectedin escalating group (escalating doses of lurbinectedin plus fixed irinotecan dose of 75 mg/m^2^) and the irinotecan D1,D8 escalating group (fixed lurbinectedin dose of 3.0 mg/m^2^ plus escalating doses of irinotecan). The third group was the irinotecan D1 escalating group, which evaluated a schedule of a fixed lurbinectedin dose of 2.6 mg/m^2^ plus escalating doses of irinotecan, both on D1 q3wk. In all groups, lurbinectedin was administered as 1-h intravenous (i.v.) infusions and irinotecan as 90-min i.v. infusions. Treatment was administered until disease progression, unacceptable toxicity, intercurrent illness precluding study continuation, patient refusal and/or non-compliance with study requirements, treatment delay > 15 days (except if with clear clinical benefit), > 2 dose reductions per drug (irinotecan or lurbinectedin), and treatment-related grade ≥ 3 rhabdomyolysis.

Regardless of escalating group, all patients received antiemetic prophylaxis before each irinotecan infusion. Patients could also receive antidiarrheal prophylaxis in the event of cholinergic syndrome, and secondary granulocyte colony-stimulating factor (G-CSF) prophylaxis. Primary G-CSF prophylaxis was not allowed at the start of dose escalation, but it could be implemented if there was an excess of dose-limiting neutropenia.

### Dose escalation and dose-limiting toxicities

Dose escalation in each group followed a standard 3 + 3 design.

In the lurbinectedin escalating group, escalation started at lurbinectedin 1.0 mg/m^2^ plus irinotecan 75 mg/m^2^. The starting dose for lurbinectedin was 50% of the body surface area (BSA)-based RD of 2.0 mg/m^2^ q3wk determined for lurbinectedin combined with doxorubicin in a previous phase Ib study [8–10]. The fixed irinotecan dose of 75 mg/m^2^ was similar to the RDs of 70–100 mg/m^2^ found for the same schedule when combined with other drugs [11–13]. In the irinotecan D1,D8 escalating group, escalation started at lurbinectedin 30 mg/m^2^ plus irinotecan 15 mg/m^2^. The fixed lurbinectedin dose of 3.0 mg/m^2^ was slightly lower that the single-agent RD of 3.2 mg/m^2^ q3wk and was chosen to ensure therapeutic activity, as irinotecan escalation started at a low dose. As for the irinotecan D1 escalating group, escalation started at lurbinectedin 2.6 mg/m^2^ plus irinotecan 75 mg/m^2^. The starting dose for lurbinectedin was 80% of the single-agent RD of 3.2 mg/m^2^, whereas the starting dose for irinotecan was 33% of the irinotecan dose of 75 mg/m^2^ evaluated in the lurbinectedin escalating group.

The following DLTs were defined for all groups: grade 4 neutropenia > 3 days; febrile neutropenia or neutropenic sepsis; grade 4 thrombocytopenia (or grade 3 requiring transfusion); grade 4 transaminase increase (or grade 3 for > 14 days); grade ≥ 2 transaminase increase with total bilirubin increase ≥ 2 × upper limit of normal and normal alkaline phosphatase; grade ≥ 3 diarrhea lasting > 5 days despite adequate corrective treatment; grade ≥ 3 creatine phosphokinase increase; any clinically relevant grade ≥ 3 toxicity; and cycle delay > 15 days or omission of the irinotecan D8 infusion in Cycle 1 due to toxicity.

### Study assessments

Patients were evaluable for efficacy if they received at least one dose each of irinotecan and lurbinectedin. Overall response rate (ORR) was the percentage of patients with confirmed response (complete or partial) as per Response Evaluation Criteria In Solid Tumors (RECIST) v.1.1 [14] and according to Investigator’s assessment (IA). Clinical benefit rate (CBR) was the percentage of patients with confirmed response or disease stabilization for ≥ 4 months. Disease control rate (DCR) was the percentage of patients with confirmed response or disease stabilization. Time-to-event parameters were duration of response (DOR), progression-free survival (PFS) and overall survival (OS).

Hematology and biochemistry tests were done at baseline, weekly during Cycle 1, and on D1 and D8 (or D1 in the irinotecan D1 escalating group) during subsequent cycles. Electrocardiograms were obtained at baseline and repeated whenever clinically indicated. AEs and laboratory abnormalities were graded with the National Cancer Institute Common Terminology Criteria for Adverse Events (NCI-CTCAE) v.4 [15], and coded using the Medical Dictionary for Regulatory Activities (MedDRA) v.25.0.

### Pharmacokinetic analyses

Twelve samples were collected from each patient to quantify lurbinectedin, irinotecan and SN-38 plasma concentrations at baseline and at different times during one week after the first treatment administration in Cycle 1. Analytes were measured by validated liquid extraction methods followed by ultra-performance liquid chromatography tandem mass-spectrometry detection (Anapharm Bioanalytics, Barcelona, Spain). The calibration ranges were 0.1–50 ng/mL for lurbinectedin, 2.5–250 ng/mL for irinotecan, and 0.1–50 ng/mL for SN-38.

### Statistical analysis

Continuous variables were presented with summary statistics, and categorical variables in frequency tables. Time-to-event variables were calculated using the Kaplan–Meier method. Binomial exact distribution was used to calculate 95% confidence intervals (CIs) for categorical variables.

## Results

### Dose escalation

Overall, 83 patients were treated with the combination in the escalation stage: 39 in the lurbinectedin escalating group, 26 in the irinotecan D1,D8 escalating group, and 18 in the irinotecan D1 escalating group. Supplementary Figure S1 shows the number of patients and the dose levels evaluated in each group. All DLTs at the highest dose level evaluated without primary G-CSF prophylaxis in each group consisted of grade 3/4 neutropenia with or without fever (Table 1). As a result, dose escalation continued in all groups but with added primary G-CSF prophylaxis after Day 1 of each cycle. For patients treated after adding primary G-CSF prophylaxis, most DLTs consisted of hematological abnormalities, including neutropenia and thrombocytopenia. In the irinotecan D1 escalating group, 2 patients died due to DLTs (one with grade 5 neutropenic colitis in Cycle 4, and one with grade 5 sepsis in Cycle 1), and therefore dose escalation in this group was discontinued (Table 1). Two RDs were defined for the combination with primary G-CSF prophylaxis: lurbinectedin 2.0 mg/m^2^ plus irinotecan 75 mg/m^2^ in the lurbinectedin escalating group, and lurbinectedin 3.0 mg/m^2^ plus irinotecan 40 mg/m^2^ in the irinotecan D1,D8 escalating group. No RDs were defined in the irinotecan D1 escalating group, or for the combination without primary G-CSF prophylaxis in the other escalating groups.

**Table 1:** Distribution of patients and dose-limiting toxicities over the dose levels and escalating groups evaluated during the phase I stage

		Dose level	Dose		No. of patients with DLTs/ No. of evaluable patients		DLT
			Lurbinectedin (mg/m^2^)	Irinotecan (mg/m^2^)			
Lurbinectedin Escalating Group (n = 39)	Without primary G-CSF prophylaxis	DL1	1.0	75	1/6		Grade 3 neutropenia preventing irinotecan administration on Day 8 (*n* = 1)
		DL2	1.5	75	1/3		Grade 3 febrile neutropenia (*n* = 1) ^a^
		DL3 (MTD)	2.0	75	Overall: 4/11	1/3	Grade 3 neutropenia preventing irinotecan administration on Day 8 (*n* = 1)
						Expansion: 3/8	Grade 3 febrile neutropenia (*n* = 2)
							Grade 4 neutropenia preventing irinotecan administration on Day 8 (*n* = 1)
	With primary G-CSF prophylaxis	DL3 (RD)	2.0	75	Overall: 3/10	0/3	
						Expansion: 3/7	Grade 3 neutropenia preventing irinotecan administration on Day 8 (*n* = 2)
							Grade 2 thrombocytopenia preventing irinotecan administration on Day 8 (*n* = 1)
		DL4 (MTD)	2.4	75	2/2		Grade 2 thrombocytopenia preventing irinotecan administration on Day 8 (*n* = 1)
							Grade 4 neutropenia preventing irinotecan administration on Day 8 (*n* = 1)
Irinotecan D1,D8 Escalating Group (n = 26)	Without primary G-CSF prophylaxis	DL1	3.0	15	Overall: 2/9	1/3	Grade 3 neutropenia preventing irinotecan administration on Day 8 (*n* = 1)
						Expansion: 1/6	Grade 3 neutropenia preventing irinotecan administration on Day 8 (*n *= 1)
	With primary G-CSF prophylaxis	DL2	3.0	30	Overall: 1/6	1/3	Grade 3 ALT increase preventing irinotecan administration on Day 8 (*n* = 1)
						Expansion: 0/3	
		DL3 (RD)	3.0	40	Overall: 1/6	1/3	Grade 3 neutropenia preventing irinotecan administration on Day 8 (*n* = 1)
						Expansion: 0/3	
		DL4 (MTD)	3.0	50	2/3		Grade 3 thrombocytopenia preventing irinotecan administration on Day 8 (*n* = 1)
							Grade 3/4 neutropenia preventing irinotecan administration on Day 8 (*n* = 1)
Irinotecan D1 Escalating Group (n = 18)	Without primary G-CSF prophylaxis	DL1 (MTD)	2.6	50	2/3		Grade 3 febrile neutropenia (*n* = 1)
							Grade 4 neutropenia (*n* = 1)
	With primary G-CSF prophylaxis	DL1	2.6	50	Overall: 1/5	0/3	
						Expansion: 1/2	Grade 4 febrile neutropenia (*n* = 1) Grade 5 sepsis (*n* = 1)
		DL2 (MTD)	2.6	60	Overall: 2/9	1/3	Grade 3 febrile neutropenia (*n* = 1) Grade 4 thrombocytopenia (*n* = 1)
						Expansion: 1/6	Grade 5 neutropenic colitis (*n* = 1) ^b^

Dose levels are shown in the same order they were evaluated

^a^Delayed-onset DLT that occurred in Cycle 3, after the decision to escalate to DL3 had been taken

^b^Delayed-onset DLT that occurred in Cycle 4

*D* Day, *DL *dose level, *DLT* dose-limiting toxicity, *G-CSF *granulocyte colony-stimulating factor, *MTD* maximum tolerated dose, *RD* recommended dose

### Characteristics of patients

Baseline characteristics of the 83 patients treated in the escalation stage are summarized in Table 2. Most common tumor types were SCLC (*n* = 26, 31%), soft tissue sarcoma (STS) (*n* = 16, 19%) and mesothelioma (*n* = 10, 12%). Most patients had locally advanced (*n* = 31, 37%) or metastatic (*n* = 41, 49%) disease at diagnosis. Thirty patients (36%) had bulky disease (defined as any target lesion > 50 mm) at baseline. Nearly all patients had received prior systemic therapy (*n* = 81, 98%).

**Table 2:** Baseline characteristics of patients treated with lurbinectedin plus irinotecan during the phase I stage

	Lurbinectedin plus Irinotecan Escalating Group				
	Lurbinectedin		Irinotecan D1,D8		Irinotecan D1
	RD (Lurbinectedin 2.0 mg/m^2^ + Irinotecan 75 mg/m^2^ + G-CSF) (*n* = 13)	All dose levels (n = 39)	RD (Lurbinectedin 3.0 mg/m^2^ + Irinotecan 40 mg/m^2^ + G-CSF) (*n* = 6)	All dose levels (*n* = 26)	All dose levels ^a^ (*n* = 18)
Gender					
Male	6 (46)	17 (44)	5 (83)	16 (62)	9 (50)
Female	7 (54)	22 (56)	1 (17)	10 (38)	9 (50)
Median age (range) (years)	59.0 (22–70)	58.0 (22–75)	60.5 (49–74)	59.0 (24–76)	64.5 (52–76)
ECOG PS					
0	5 (38)	10 (26)	3 (50)	15 (58)	5 (28)
1	8 (62)	29 (74)	3 (50)	11 (42)	13 (72)
Median BSA (range) (m^2^)	1.8 (1.4–2.1)	1.8 (1.4–2.4)	1.8 (1.6–2-0)	1.8 (1.3–2.5)	1.8 (1.5–2.2)
Median albumin (range) (g/dL)	4.3 (3.0–5.0)	4.1 (3.0–5.0)	4.3 (3.6–4.7)	4.2 (3.0–4.7)	4.1 (3.5–4.7)
Primary tumor					
SCLC	4 (31)	11 (28)	3 (50)	5 (19)	10 (56)
STS	2 (15)	8 (21)	1 (17)	7 (27)	1 (6)
Leiomyosarcoma	1 (8)	3 (8)	1 (17)	3 (12)	
Chondrosarcoma		1 (3)		1 (4)	
Chordoma	1 (8)	1 (3)		1 (4)	
Ewing sarcoma		1 (3)		1 (4)	
Carcinosarcoma		1 (3)			
Myoepithelial carcinoma		1 (3)			
Spindle cell sarcoma					1 (6)
Synovial sarcoma				1 (4)	
Mesothelioma		5 (13)	2 (33)	5 (19)	
Endometrial carcinoma	2 (15)	2 (5)			7 (39)
Pancreatic adenocarcinoma	1 (8)	5 (13)		4 (15)	
Glioblastoma	2 (15)	2 (5)		2 (8)	
CRC		3 (8)			
Epithelial ovarian carcinoma	2 (15)	3 (8)			
Gastric carcinoma				2 (8)	
GEP-NET				1 (4)	
Stage at diagnosis					
Early	5 (38)	7 (18)	1 (17)	2 (8)	2 (11)
Locally advanced	4 (31)	15 (38)	1 (17)	10 (38)	6 (33)
Metastatic	4 (31)	17 (44)	4 (67)	14 (54)	10 (56)
Median no. of sites of disease involvement at baseline (range)	3.0 (1–4)	3.0 (1–8)	3.0 (1–5)	3.0 (1–5)	3.0 (1–4)
Bulky disease at baseline ^b^	3 (23)	16 (41)	2 (33)	7 (27)	7 (39)
Median TTP of last prior therapy (range) (months)	6.0 (1.4–19.5)	5.4 (1.1–23.8)	4.6 (1.1–7.6)	5.8 (0.1–19.1)	6.6 (1.8–13.1)
Prior medical anticancer therapy	13 (100)	39 (100)	5 (83) ^c^	24 (92) ^c^	18 (100)
Median no. of prior anticancer therapies (range)	2.0 (1–7)	2.0 (1–7)	1.0 (0–2)	1.5 (0–3)	2.0 (1–3)
Prior radiotherapy	9 (69)	21 (54)	4 (67)	15 (58)	10 (56)
Prior surgery	7 (54)	17 (44)	2 (33)	13 (50)	7 (39)

Data are n (%) of patients, except for median (range)

^a^No RD was defined for this dose escalation group

^b^Defined as any target lesion > 50 mm

^c^Two patients in this escalation group underwent prior surgery with or without radiotherapy but received no prior systemic therapy

*BSA* body surface area, *CRC* colorectal cancer, *D* Day, *ECOG* Eastern Cooperative Oncology Group, *G-CSF *granulocyte colony-stimulating factor, *GEP-NET* gastroenteropancreatic neuroendocrine tumors, *RD* recommended dose, *SCLC* small cell lung cancer,* STS* soft tissue sarcoma, *TTP* time to progression

### Treatment administration

Exposure to lurbinectedin/irinotecan in the escalation stage is shown in Table 3.

**Table 3:** Exposure to treatment and reasons for treatment discontinuation at each escalating group

	Lurbinectedin plus Irinotecan Escalating Group				
	Lurbinectedin		Irinotecan D1,D8		Irinotecan D1
	RD (Lurbinectedin 2.0 mg/m^2^ + Irinotecan 75 mg/m^2^ + G-CSF) (n = 13)	All dose levels (n = 39)	RD (Lurbinectedin 3.0 mg/m^2^ + Irinotecan 40 mg/m^2^ + G-CSF) (n = 6)	All dose levels (n = 26)	All dose levels ^a^ (n = 18)
Median no. of cycles administered per patient (range)	6.0 (1–43)	4.0 (1–43)	5.0 (2–16)	4.0 (1–76)	6.0 (1–42)
Patients treated with					
≥ 6 cycles	8 (62)	17 (44)	3 (50)	10 (38)	9 (50)
≥ 12 cycles	2 (15)	5 (13)	1 (17)	4 (15)	4 (22)
Median time on treatment, months (range)	5.0 (0.8–30.7)	3.8 (0.5–30.7)	3.9 (0.9–11.5)	3.2 (0.9–56.6)	4.4 (0.2–30.4)
Median relative dose intensity, % (range)					
Lurbinectedin	95.5 (72.9–103.0)	97.5 (58.6–103.0)	98.7 (93.3–100.3)	97.8 (76.9–104.8)	94.8 (63.7–101.8)
Irinotecan	75.2 (45.6–100.0)	87.0 (45.6–102.3)	81.0 (51.8–98.1)	80.1 (50.0–104.4)	47.7 (33.5–51.3)
Patients with					
Treatment-related cycle delays	5 (38)	14 (36)	1 (17)	4 (15)	4 (22)
Treatment-related irinotecan dose omissions	8 (62)	20 (51)	2 (33)	11 (42)	. ^b^
Treatment-related lurbinectedin and/or irinotecan dose reductions	3 (23)	8 (21)	2 (33)	10 (38)	3 (17)
Reasons for treatment discontinuation					
Progressive disease	10 (77)	30 (77)	4 (67)	22 (84)	12 (67)
Patient refusal	2 (15)	3 (8)		2 (8)	
Treatment-related AE	1 (8)	2 (5) ^c^			4 (22) ^d^
Death ^e^		2 (5)	2 (33)	2 (8)	1 (6)
Investigator’s decision		1 (3)			1 (6)
Other		1 (3) ^f^			

Data are n (%) of patients, except for median (range)

^a^No RD was defined for this group

^b^No irinotecan dose omissions occurred in this group, as these were not possible with a D1 q3wk schedule

^c^ALT/AST increase with bilirubin increase (*n* = 1, at the RD), and anemia (*n *= 1)

^d^Thrombocytopenia alone (*n* = 1) or concomitant with febrile neutropenia (*n* = 1); neutropenic colitis (*n* = 1); and sepsis (*n* = 1). The neutropenic colitis and sepsis also resulted in the death of the patient

^e^All deaths in this category were due to non treatment-related AEs or disease progression

^f^Investigator’s decision due to deterioration of the patient’s condition

AE, adverse event; ALT, alanine aminotransferase; ASP, aspartate aminotransferase; D, Day; G-CSF, granulocyte colony-stimulating factor; RD, recommended dose; q3wk, every there weeks

In the lurbinectedin escalating group, 13 patients were treated at the RD of lurbinectedin 2.0 mg/m^2^ plus irinotecan 75 mg/m^2^ with primary G-CSF prophylaxis and received a median of 6.0 cycles per patient (range, 1–43 cycles), with 8 (62%) and 2 (15%) patients receiving at least 6 and 12 cycles, respectively. Patients were on treatment for a median of 5.0 months (range, 0.8–30.7 months). Median relative dose intensity was 95.5% (range, 72.9–103.0%) for lurbinectedin and 75.2% (range, 45.6–100%) for irinotecan. Most patients (n = 10, 77%) discontinued treatment due to disease progression; one patient (8%) with glioblastoma discontinued owing to treatment-related grade 4 alanine aminotransferase (ALT)/aspartate aminotransferase (AST) increase concomitant with grade 2 bilirubin increase after 7 cycles.

In the irinotecan D1,D8 escalating group, 6 patients were treated at the RD of lurbinectedin 3.0 mg/m^2^ plus irinotecan 40 mg/m^2^ with primary G-CSF prophylaxis and received fewer cycles than the other RD: a median of 5.0 cycles per patient (range, 2–16 cycles), with 3 (50%) and 1 (17%) patient receiving at least 6 and 12 cycles, respectively. Median relative dose intensity was 98.7% (range, 93.3–100.3%) for lurbinectedin and 81.0% (range, 51.8–98.1%) for irinotecan. Disease progression (n = 4, 67%) was the most common reason for treatment discontinuation; no treatment-related discontinuations occurred at this RD.

At all dose levels in the irinotecan D1 escalating group (*n* = 18 patients), patients received a median of 6.0 cycles (range, 1–42 cycles) each. Median relative dose intensity was 94.8% (range, 63.7–101.8%) for lurbinectedin and 47.7% (range, 33.5–51.3%) for irinotecan. Disease progression (*n* = 12, 67%) was the most common reason for treatment discontinuation. Four patients (22%) discontinued due to treatment-related AEs (see footnote in Table 3).

### Safety

All 83 treated patients were evaluable for safety. The most frequent treatment-related AEs or with unknown relationship at the two RDs of the lurbinectedin and irinotecan D1,D8 escalating groups, and at all dose levels in the irinotecan D1 escalating group, are shown in Table 4.

**Table 4:** Treatment-related adverse events and laboratory abnormalities regardless of relationship (> 10% of patients or grade ≥ 3) at the recommended doses of the lurbinectedin and irinotecan D1,D8 escalating groups, and at all dose levels in the irinotecan D1 escalating group

	Lurbinectedin plus Irinotecan Escalating Group									
	Lurbinectedin			Irinotecan D1,D8			Irinotecan D1			
	RD (Lurbinectedin 2.0 mg/m^2^ + Irinotecan 75 mg/m^2^ + G-CSF) (*n* = 13)			RD (Lurbinectedin 3.0 mg/m^2^ + Irinotecan 40 mg/m^2^ + G-CSF) (*n* = 6)			All dose levels (*n *= 18)			
NCI-CTCAE grade	Total	3	4	Total	3	4	Total	3	4	5
Hematological laboratory abnormalities										
Anemia	13 (100)	3 (23)		6 (100)	2 (33)		17 (94)	8 (44)		
Neutropenia	10 (77)	3 (23)	2 (15)	3 (50)	1 (17)	2 (33)	13 (72)	3 (17)	8 (44)	
Thrombocytopenia	5 (39)			3 (50)			12 (67)	4 (22)	1 (6)	
Biochemical laboratory abnormalities										
ALT increased	4 (31)		1 (8)	2 (33)			13 (72)			
AP increased	7 (54)			2 (33)			10 (59)			
AST increased	5 (39)		1 (8)	2 (33)			12 (67)	1 (6)		
Creatinine increased	12 (92)			5 (83)			17 (94)	2 (11)		
Treatment-related adverse events										
Abdominal hernia				1 (17)						
Abdominal pain	2 (15)									
Abdominal pain upper	1 (8)			1 (17)			3 (17)			
Alopecia	1 (8)			1 (17)			1 (6)			
Arthralgia							2 (11)			
Constipation	4 (31)						3 (17)			
Decreased appetite	9 (69)			3 (50)			6 (33)			
Diarrhea	6 (46)	2 (15)		3 (50)			12 (67)			
Dysgeusia	2 (15)						2 (11)			
Extravasation				1 (17)						
Fatigue	10 (77)	1 (8)		3 (50)			13 (72)			
Febrile neutropenia							3 (17)	1 (6)	2 (11)	
Hyperhidrosis	1 (8)			1 (17)						
Mucosal inflammation	2 (15)						2 (11)			
Nail dystrophy				1 (17)						
Neutropenic colitis							1 (6)			1 (6)
Nausea	11 (85)			4 (67)			8 (44)			
Phlebitis	1 (8)			1 (17)						
Pneumonitis							1 (6)	1 (6)		
Pyrexia	2 (15)						2 (11)			
Sepsis							1 (6)			1 (6)
Superficial vein thrombosis				1 (17)						
Vomiting	7 (54)			1 (17)			5 (28)			

Data are n (%) of patients

Hematological and biochemical abnormalities are shown regardless of relationship to treatment

*ALT* alanine aminotransferase, *AP *alkaline phosphatase, *AST* aspartate aminotransferase, *D* Day, *G-CSF *granulocyte colony-stimulating factor, *NCI-CTCAE *National Cancer Institute Common Terminology Criteria for Adverse Events

Most treatment-related non-hematological AEs at the RD of lurbinectedin 2.0 mg/m^2^ plus irinotecan 75 mg/m^2^ with primary G-CSF prophylaxis (*n* = 13 patients) were grade 1 or 2. The most common were gastrointestinal disorders (nausea [*n* = 11, 85%], vomiting [*n* = 7, 54%], diarrhea [n = 6, 46%]), fatigue (*n* = 10, 77%) and decreased appetite (*n* = 9, 69%). Severe non-hematological AEs were grade 3 and comprised diarrhea (*n* = 2, 15%) and fatigue (*n* = 1, 8%). Hematological laboratory abnormalities regardless of relationship were common, with severe abnormalities comprising grade ≥ 3 neutropenia (*n* = 5, 38%; grade 4 in *n* = 2, 15%) and grade 3 anemia (*n* = 3, 23%). Support requirements while on treatment comprised red blood cells (RBC) transfusions (*n* = 4, 31%), erythropoiesis-stimulating agents (*n* = 2, 15%), platelet transfusions (*n* = 1, 8%), and therapeutic use of G-CSF (*n* = 1, 8%). Most biochemical laboratory abnormalities regardless of relationship were grade 1/2; the only severe abnormality was the aforementioned episode of grade 4 ALT/AST increase that resulted in treatment discontinuation.

All treatment-related non-hematological AEs at the RD of lurbinectedin 3.0 mg/m^2^ plus irinotecan 40 mg/m^2^ with primary G-CSF prophylaxis (*n* = 6 patients) were grade 1/2. The most frequent were gastrointestinal disorders (nausea [*n* = 4, 67%], diarrhea [*n* = 3, 50%]), fatigue (*n* = 3, 50%) and decreased appetite (*n* = 3, 50%). Severe hematological laboratory abnormalities regardless of relationship comprised grade ≥ 3 neutropenia (*n* = 3, 50%; grade 4 in *n* = 2, 33%) and grade 3 anemia (*n* = 2, 33%). RBC transfusions and erythropoiesis-stimulating agents were given to 1 (17%) patient each. All biochemical laboratory abnormalities were grade 1/2.

At all dose levels in the irinotecan D1 escalating group (*n* = 18 patients), the most frequent treatment-related non-hematological AEs were fatigue (*n* = 13, 72%), gastrointestinal disorders (diarrhea [*n* = 12, 67%], nausea [*n* = 8, 44%], vomiting [*n* = 5, 28%]) and decreased appetite (*n* = 6, 33%). Severe non-hematological AEs comprised grade 3/4 febrile neutropenia (*n* = 3, 17%), grade 3 pneumonitis, grade 5 neutropenic colitis, and grade 5 sepsis (*n* = 1, 6% each).The most common severe hematological abnormalities regardless of relationship were grade ≥ 3 neutropenia (*n* = 11, 61%; grade 4 in n = 8, 44%), grade 3 anemia (*n* = 8, 44%) and grade ≥ 3 thrombocytopenia (*n* = 5, 28%; grade 4 in *n* = 1, 6%). Due to these abnormalities, 7 patients (39%) required RBC transfusions, 3 patients (17%) required therapeutic use of G-CSF, 2 patients (11%) were given EPO and 1 patient (5.6%) required platelet transfusions. Severe biochemical abnormalities comprised grade 3 creatinine increase (*n* = 2, 11%) and grade 3 AST increase (*n* = 1, 6%).

No deaths in the lurbinectedin and irinotecan D1,D8 escalating groups were related to treatment or had an unknown relationship. Two deaths (11% of patients; one each due to neutropenic colitis and sepsis) at all dose levels in the irinotecan D1 escalating group were due to treatment-related AEs.

### Efficacy

All 83 treated patients were evaluable for efficacy. Best response per RECIST is provided in Table 5.

**Table 5:** Best tumor response according to Response Evaluation Criteria In Solid Tumors (RECIST) at each escalating group

		Lurbinectedin plus Irinotecan Escalating Group				
		Lurbinectedin		Irinotecan D1,D8		Irinotecan D1
		RD (Lurbinectedin 2.0 mg/m^2^ + Irinotecan 75 mg/m^2^ + G-CSF) (*n* = 13)	All dose levels (*n* = 39)	RD (Lurbinectedin 3.0 mg/m^2^ + Irinotecan 40 mg/m^2^ + G-CSF) (*n *= 6)	All dose levels (*n* = 26)	All dose levels (*n* = 18)
PR		4 (31)	5 (13)	1 (17)	2 (8)	6 (33)
SD	≥ 4 mo	5 (38)	13 (33)	1 (17)	7 (27)	4 (22)
	< 4 mo	1 (8)	9 (23)	3 (50)	7 (27)	3 (17)
PD		3 (23)	7 (18)		8 (31)	3 (17)
Inevaluable			5 (13)	1 (17)	2 (8)	2 (11)
ORR, % ^a^ (95% CI)		30.8 (9.1–61.4)	12.8 (4.3–27.4)	16.7 (0.4–64.1)	7.7 (0.9–25.1)	33.3 (13.3–59.0)
CBR, % ^b^ (95% CI)		69.2 (38.6–90.9)	46.2 (30.1–62.8)	33.3 (4.3–77.7)	34.6 (17.2–55.7)	55.6 (30.8–78.5)
DCR, % ^c^ (95% CI)		76.9 (46.2–95.0)	69.2 (52.4–83.0)	83.3 (35.9–99.6)	61.5 (40.6–79.8)	72.2 (46.5–90.3)

Data are n (%) of patients, except for ORR, CBR and DCR

^d^Defined as percentage of patients with response

^e^Defined as percentage of patients with response or SD ≥ 4 months

^f^Defined as percentage of patients with response or SD

*CI* confidence interval, *CBR* clinical benefit rate, *DCR* disease control rate, *mo* months, *ORR* overall response rate, *PD* progressive disease, *PR* partial response, *RD* recommended dose, *SCLC* small cell lung cancer, *SD* stable disease

In the lurbinectedin escalating group, antitumor activity consisted of 5 confirmed PRs and 22 stable diseases (SD) (of which 13 had prolonged SD [defined as duration ≥ 4 months] and 9 had SD < 4 months) in 39 evaluable patients at all dose levels. Four PRs, 5 SD ≥ 4 months and 1 SD < 4 months occurred in 13 evaluable patients at the RD of lurbinectedin 2.0 mg/m^2^ plus irinotecan 75 mg/m^2^ with primary G-CSF prophylaxis (ORR = 30.8%; CBR = 69.2%) (Table 5). The tumor types with confirmed PR were SCLC (*n* = 3) and endometrial carcinoma (*n* = 2), while those with SD ≥ 4 months were SCLC (*n *= 5), STS (*n* = 5, including leiomyosarcoma [*n* = 2], chordoma, Ewing sarcoma, and myoepithelial carcinoma [*n* = 1 each]), glioblastoma (*n* = 2) and pancreatic adenocarcinoma (*n* = 1). Objective tumor shrinkage was observed in 19 (55.9%) of 34 patients with at least one radiological tumor assessment at all dose levels, and in 7 (53.8%) of 13 patients at the RD (Supplementary Figure S2A).

In the irinotecan D1,D8 escalating group there were 2 confirmed PRs and 14 SD (of which 7 had SD ≥ 4 months and 7 had SD < 4 months) in 26 evaluable patients at all dose levels. One PR, 1 SD ≥ 4 months and 3 SD < 4 months were found in 6 evaluable patients at the RD of lurbinectedin 3.0 mg/m^2^ plus irinotecan 40 mg/m^2^ with primary G-CSF prophylaxis (ORR = 16.7%; CBR = 33.3%) (Table 5). The confirmed PRs were observed in one patient each with SCLC and STS (synovial sarcoma), while the 7 SD ≥ 4 months occurred in patients with STS (*n* = 3, including chondrosarcoma, Ewing sarcoma and leiomyosarcoma [*n* = 1 each]), pancreatic adenocarcinoma (*n* = 2), glioblastoma and mesothelioma (n = 1 each). Thirteen (54.2%) of 24 patients with at least one radiological tumor assessment at all dose levels showed tumor shrinkage, including 3 (60.0%) of 5 patients at the RD (Supplementary Figure S2B).

Finally, 6 confirmed PRs and 7 SD (including 4 SD ≥ 4 months and 3 SD < 4 months) occurred in 18 evaluable patients at all dose levels of the irinotecan D1 escalating group (Table 5). The tumor types with confirmed PR in this group were SCLC (*n* = 4) and endometrial carcinoma (*n* = 2), while those with SD ≥ 4 months were SCLC (*n* = 2), endometrial carcinoma and STS (spindle cell sarcoma) (*n* = 1 each). Eleven (68.8%) of 16 patients with at least one radiological tumor assessment in this group showed tumor shrinkage (Supplementary Figure S2C).

Overall, 5 PRs and 10 SD (including 6 SD ≥ 4 months and 4 SD < 4 months) occurred in the 19 patients treated at the two RDs defined in this stage. Ten of these patients (55.6% of 18 patients with radiological tumor assessments) showed tumor shrinkage (Supplementary Figure S2D), and their tumor types were SCLC (*n* = 5), STS (*n* = 2), endometrial carcinoma (n = 2) and glioblastoma (n = 1).

### Pharmacokinetics

Plasma samples for non-compartmental analysis (NCA) of concentrations of lurbinectedin, irinotecan and SN-38 were collected from all patients. Parameters obtained during Cycle 1 at each dose level are shown in Supplementary Table S1. Wide variability was observed in total clearance (CL) for all three analytes. No major differences in lurbinectedin CL were observed according to lurbinectedin dose level, or in irinotecan and SN-38 CL according to irinotecan dose level, thereby confirming dose-proportionality of the analytes. An assessment of the relationships between the CL and area under the concentration–time curve (AUC) showed high inter-individual variability of CL regardless of the AUC of other analytes, and linear regression lines with negative slopes (Supplementary Figure S3).

## Discussion

The first part of this phase I/II study defined two RDs for lurbinectedin on D1 plus irinotecan on D1,D8 q3wk: lurbinectedin 2.0 mg/m^2^ plus irinotecan 75 mg/m^2^, and lurbinectedin 3.0 mg/m^2^ plus irinotecan 40 mg/m^2^. Importantly, both RDs required primary G-CSF prophylaxis. An RD for the schedule of D1 Irinotecan was not defined, as it was found to be clinically unfeasible after the occurrence of two toxic deaths among 14 patients regardless of primary G-CSF usage.

The safety profile of the combination at the RDs was predictable and manageable. Myelosuppression, particularly severe neutropenia, was the most common DLT. Severe hematological abnormalities were more common at the RD of lurbinectedin 3.0 mg/m^2^ plus irinotecan 40 mg/m^2^ (anemia 33%, neutropenia 50%) than at the RD of lurbinectedin 2.0 mg/m^2^ plus irinotecan 75 mg/m^2^ (23% and 38%, respectively) or with single-agent lurbinectedin (17% and 41%, respectively). Compared with the safety profile of single-agent lurbinectedin [16], both RDs of the combination showed higher incidences of treatment-related nausea (85% and 67% *vs.* 51%), decreased appetite (69% and 50% *vs.* 17%) and diarrhea (46% and 50% *vs.* 13%). Patients treated at the RD of lurbinectedin 2.0 mg/m^2^ plus irinotecan 75 mg/m^2^ also showed higher incidences of treatment-related fatigue (77% *vs.* 53%), vomiting (54% *vs.* 25%) and constipation (31% *vs.* 17%) compared to single-agent lurbinectedin. Differences compared to single-agent lurbinectedin were consistent with the addition of irinotecan, a known inducer of fatigue, colitis and neutropenia [17–20].

The clinical data described herein showed promising antitumor activity with the combination in several indications. Antitumor activity was observed in all escalation groups and comprised 13 confirmed PRs and 24 disease stabilizations for ≥ 4 months at all groups and dose levels, including the RDs. Evidence of objective tumor shrinkage was also observed. Antitumor activity was enriched among patients with SCLC, STS (synovial sarcoma), endometrial carcinoma, glioblastoma and pancreatic adenocarcinoma.

The PK analysis confirmed dose proportionality for lurbinectedin, irinotecan and the irinotecan metabolite SN-38 across all dose levels. The PK parameters of the three analytes were in line with single-agent values reported in the literature [21–23], thereby ruling out major PK drug-drug interactions between lurbinectedin and irinotecan.

In conclusion, the escalation stage of this trial showed that a schedule of lurbinectedin on D1 combined with irinotecan on D1,D8 q3wk is feasible and has a manageable overall safety profile at the two defined RDs in patients with selected advanced solid tumors. Antitumor activity with the combination comprised objective PRs as per RECIST v.1.1, prolonged disease stabilizations, and evidence of objective tumor shrinkage. Compared to this schedule, a higher response rate was observed with a schedule of lurbinectedin and irinotecan both on D1 q3wk, but owing to the latter’s safety profile no RD could be defined. The RD defined in the lurbinectedin escalating group (i.e., lurbinectedin 2.0 mg/m^2^ plus irinotecan 75 mg/m^2^ with primary G-CSF prophylaxis) was selected for further evaluation in the phase II stage of the trial, based on its safety profile and the finding of efficacy in different tumors. The tumor types chosen for evaluation in this stage were SCLC, synovial sarcoma, endometrial carcinoma, glioblastoma and neuroendocrine tumors (the latter were chosen based on prior experience with lurbinectedin in pretreated patients with small cell lung neuroendocrine carcinoma, both as single-agent and in combination with other chemotherapeutic agents) [6, 8, 24]. The lurbinectedin/irinotecan combination is also being evaluated in an ongoing randomized phase III study (LAGOON trial; ClinicalTrials.gov identifier: NCT05153239), which is comparing lurbinectedin alone or combined with irinotecan *vs.* topotecan or irinotecan alone in second-line SCLC after failure of one prior platinum-containing chemotherapy [25].

## Supplementary Information

Below is the link to the electronic supplementary material.

Supplementary file 1 (DOCX 366 KB)

## Data Availability

No datasets were generated or analysed during the current study.
