# Supplementary material for: Phase I results on the efficacy, safety and pharmacokinetics of lurbinectedin and irinotecan in advanced solid tumors
Source: Invest New Drugs. 2025 Sep 18;43(4):955–67. doi: 10.1007/s10637-025-01583-y (PMC12515209; doi:10.1007/s10637-025-01583-y)
Supplement: Supplementary file 1 — (DOCX 366 KB) [file 10637_2025_1583_MOESM1_ESM.docx]

Supplementary information

**Article title:** Phase I Results on the Efficacy, Safety and Pharmacokinetics of Lurbinectedin and Irinotecan in Advanced Solid Tumors

**Journal name:** Investigational New Drugs.

**Author names:** Alejandro Falcón^1^, Santiago Ponce^2,3,4^, Gregory M. Cote^5^, Ana Gil^1^, Jessica J. Lin^5^, Bruno Bockorny^6^, Julia Martínez^1^, Carmen Kahatt^7^, Sara Martinez^7^, Pablo Zubiaur^7^, Mariano Siguero^7^, Martin Cullell-Young^7^, Javier Jiménez^7^, Jon Zugazagoitia^2,3,4^. and Luis Paz-Ares^2,3,4,8^.

**Author affiliation:** 1. Hospital Universitario Virgen del Rocio, Seville, Spain**.**

2. Hospital Universitario 12 de Octubre, Madrid, Spain.

3. CNIO-H12O Lung Cancer Clinical Cancer Research Unit, Instituto de Investigación Biomédica i+12 & Centro Nacional de Investigaciones Oncológicas (CNIO), Madrid, Spain.

4. CIBERONC, Madrid, Spain.

5. Mass General Cancer Center, Boston, USA.

6. Beth Israel Deaconess Medical Center, Boston, USA.

7. Pharma Mar, Colmenar Viejo, Madrid, Spain.

8. Universidad Complutense, Madrid, Spain.

**Corresponding author:** Alejandro Falcón, M.D.

E-mail: afalconglez@gmail.com

**Supplementary Information**

**Supplementary Table S1.** Non-compartmental pharmacokinetic parameters of lurbinectedin, irinotecan and SN-38 by dose level.

|  | **Lurbinectedin (mg/m^2^) / irinotecan (mg/m^2^) dose level** | | | | | | | | | | | |
| --- | --- | --- | --- | --- | --- | --- | --- | --- | --- | --- | --- | --- |
|  | **Lurbinectedin D1 Escalation**  **plus Irinotecan D1,D8** | | | |  | **Lurbinectedin D1 plus Irinotecan D1,D8 Escalation** | | | |  | **Lurbinectedin plus Irinotecan D1 Escalation** | |
|  | **1.0 / 75**  (n=6) | **1.5 / 75**  (n=4) | **2.0 / 75**  (n=25) | **2.4 / 75**  (n=4) |  | **3.0 / 15**  (n=11) | **3.0 / 30**  (n=6) | **3.0 / 40**  (n=6) | **3.0 / 50**  (n=3) |  | **2.6 / 50**  (n=8) | **2.6 / 60**  (n=10) |
| **Lurbinectedin** | | | | | | | | | | | | |
| AUC  (ng·h/mL) | 287.06  (97.60) | 309.03  (99.93) | 341.19  (243.65) | 668.45  (191.36) |  | 552.22  (550.65) | 319.36  (384.65) | 291.68  (110.92) | 633.94  (100.71) |  | 545.60  (260.29) | 707.22  (563.60) |
| CL  (L/h) | 5.95  (5.03) | 8.86  (5.07) | 11.33  (5.56) | 5.88  (1.65) |  | 9.93  (4.61) | 16.88  (9.55) | 19.09  (5.60) | 7.39  (1.29) |  | 8.21  (5.78) | 6.58  (20.39) |
| C_max_  (ng/mL) | 47.90  (17.90) | 63.75  (20.61) | 83.50  (25.32) | 125.10  (207.69) |  | 140.00  (57.52) | 82.70  (40.83) | 84.65  (35.11) | 138.00  (53.56) |  | 128.50  (51.99) | 178.00  (76.15) |
| HL  (h) | 34.92  (62.20) | 40.14  (18.08) | 47.17  (30.32) | 28.68  (9.61) |  | 47.32  (16.29) | 52.34  (22.29) | 24.49  (20.70) | 37.25  (45.77) |  | 40.64  (17.75) | 43.48  (20.39) |
| V_ss_  (L) | 282.23  (205.46) | 227.82  (128.50) | 352.36  (400.37) | 200.58  (112.79) |  | 282.66  (191.26) | 613.25  (384.87) | 338.78  (219.24) | 198.55  (433.97) |  | 198.49  (161.91) | 195.18  (146.59) |
| V_z_  (L) | 487.34  (388.48) | 503.58  (162.50) | 757.45  (750.13) | 273.12  (102.68) |  | 567.65  (408.14) | 1129.78  (501.24) | 755.16  (490.89) | 381.13  (691.44) |  | 383.74  (363.01) | 397.34  (239.88) |
| **Irinotecan** | | | | | | | | | | | | |
| AUC  (ng·h/mL) | 4780.67  (1677.12) | 4212.18  (1019.62) | 3660.94  (1150.48) | 4517.96  (482.52) |  | 678.88  (431.26) | 1245.39  (670.34) | 1663.94  (552.45) | 2342.58  (592.38) |  | 2733.46  (1020.72) | 3935.42  (1417.03) |
| CL  (L/h) | 25.89  (7.63) | 32.62  (7.96) | 35.77  (9.49) | 29.56  (5.45) |  | 44.19  (22.12) | 40.83  (18.74) | 40.36  (13.97) | 42.09  (12.89) |  | 35.56  (12.20) | 27.02  (7.25) |
| C_max_  (ng/mL) | 814.05  (147.94) | 740.24  (34.29) | 793.16  (195.91) | 951.82  (96.15) |  | 153.06  (42.25) | 247.68  (65.84) | 364.35  (87.76) | 526.65  (144.86) |  | 630.00  (142.02) | 697.41  (149.67) |
| HL  (h) | 9.02  (6.77) | 11.48  (7.37) | 8.28  (4.12) | 5.28  (3.11) |  | 6.29  (2.34) | 7.12  (3.36) | 6.60  (1.70) | 9.13  (0.89) |  | 8.75  (2.19) | 8.46  (4.38) |
| V_ss_  (L) | 248.13  (98.07) | 372.03  (79.86) | 260.15  (76.09) | 196.68  (64.45) |  | 225.12  (66.70) | 285.35  (94.70) | 269.41  (115.11) | 356.95  (124.85) |  | 259.92  (110.74) | 236.57  (59.12) |
| V_z_  (L) | 379.58  (370.26) | 559.46  (233.52) | 397.42  (145.91) | 253.32  (115.91) |  | 252.25  (112.54) | 418.14  (150.09) | 378.18  (175.18) | 487.88  (164.88) |  | 372.82  (182.96) | 317.14  (127.74) |
| **SN-38** | | | | | | | | | | | | |
| AUC  (ng·h/mL) | 274.56  (63.86) | 180.54  (93.51) | 181.96  (80.82) | 192.72  (61.06) |  | 72.23  (47.63) | 133.16  (105.69) | 156.53  (89.05) | 101.35  (71.48) |  | 87.48  (170.46) | 172.37  (104.03) |
| CL  (L/h) | 494.70  (121.99) | 831.46  (1482.38) | 776.95  (353.65) | 726.71  (184.57) |  | 415.33  (209.08) | 418.88  (269.55) | 517.24  (926.75) | 651.21  (238.21) |  | 937.32  (603.46) | 639.86  (233.83) |
| C_max_  (ng/mL) | 22.02  (3.62) | 10.20  (2.59) | 15.40  (6.53) | 24.85  (13.77) |  | 8,45  (3.20) | 11.01  (6.25) | 12.66  (4.54) | 10.21  (3.13) |  | 12.28  (16.17) | 13.11  (9.30) |
| HL  (h) | 17.94  (6.47) | 28.42  (23.77) | 16.41  (8.32) | 9.84  (6.76) |  | 10.27  (6.50) | 17.11  (6.52) | 13.58  (8.92) | 24.42  (9.08) |  | 11.49  (8.76) | 18.84  (6.59) |
| V_ss_  (L) | 10951.62  (4421.73) | 21697.58  (15818.66) | 16115.92  (6228.33) | 6773.67  (5955.84) |  | 5021.07  (1952.45) | 7734.20  (4463.14) | 7890.10  (1060.34) | 12032.75  (12689.04) |  | 15347.39  (7588.94) | 11332.05  (5598.04) |
| V_z_  (L) | 12478.52  (5891.99) | 23551.74  (22079.27) | 18724.01  (6855.21) | 7219.38  (6649.17) |  | 5193.63  (2330.62) | 7778.35  (7844.05) | 9051.33  (1290.22) | 15673.00  (13854.11) |  | 15536.71  (9403.53) | 12188.44  (6200.05) |
| Values are expressed as mean (standard deviation).  Dose levels are shown regardless of administration of primary G-CSF prophylaxis.  AUC, area under the concentration-time curve from time zero to infinity; CL, total clearance; C_max_, maximum concentration; D, Day; DL, dose level; G-CSF, granulocyte colony-stimulating factor; HL, terminal half-life; V_ss_, volume of distribution at steady-state; V_z_, apparent volume of distribution during terminal phase. | | | | | | | | | | | | |

**Supplementary Figure S1.** Dose escalating groups, dose levels, and numbers of patients treated at each dose level of lurbinectedin plus irinotecan in the phase I stage.

**
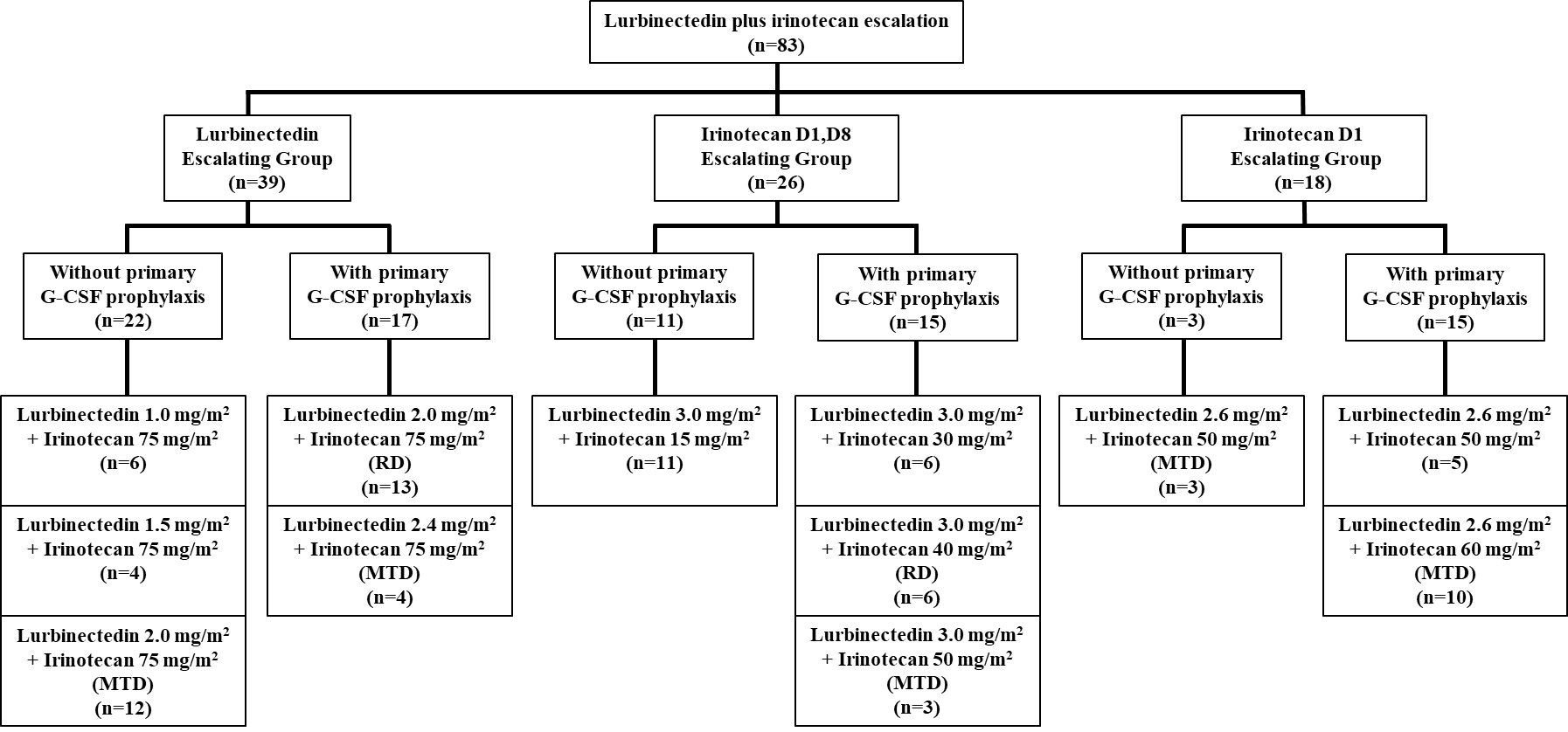
**

**Supplementary Figure S2.** Maximum variation of target lesions in patients with measurable disease treated in each escalating group and with at least one radiological tumor assessment. A: Lurbinectedin escalating group (n=34 patients). B: Irinotecan D1,D8 escalating group (n=24 patients). C: Irinotecan D1 escalating group (n=16 patients). D: Patients treated at the RD in the lurbinectedin and the irinotecan D1,D8 escalating groups. STS subtypes were (a) leiomyosarcoma, and (b) chondroid chordoma. D, Day; G-CSF, granulocyte colony-stimulating factor; MTD, maximum tolerated dose; RD, recommended dose; SCLC, small cell lung cancer; STS, soft tissue sarcoma.

**
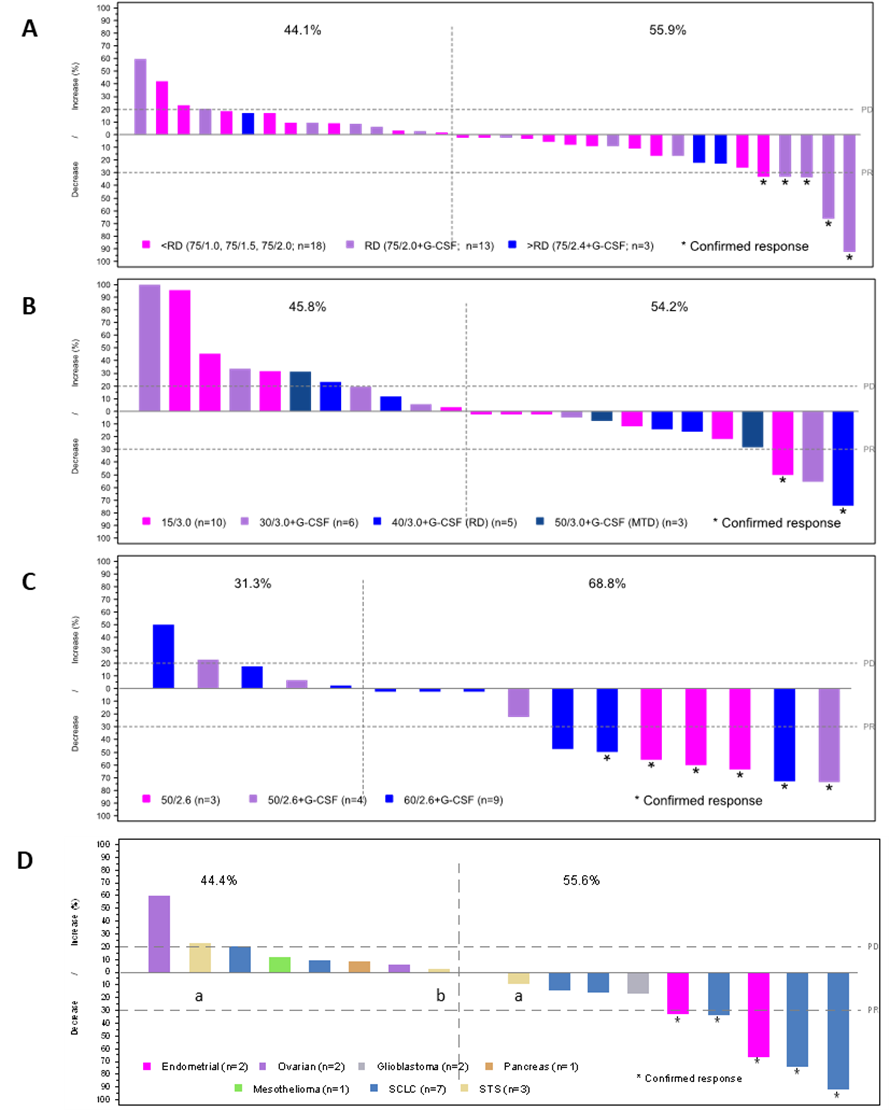
**

**Supplementary Figure S3.** Relationship between total plasma clearance and area under the concentration-time curve of different analytes. The blue lines represent the linear regression.

**
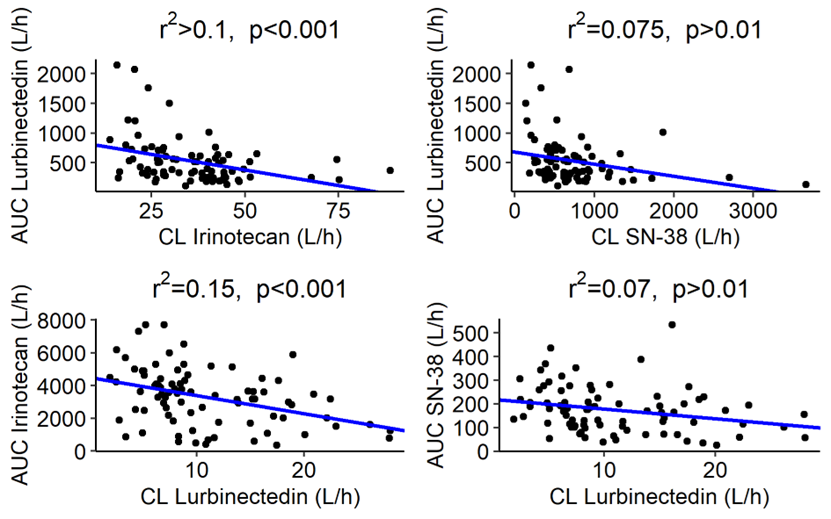
**

AUC, area under the concentration-time curve; CL, total plasma clearance; p, p-value; r2, coefficient of determination.
